# What Is Truth?

**Published:** 1917-03-17

**Authors:** 


### The Hospital

The Workers' Newspaper of Administrative Medicine and Institutional Life, Administration, National Insurance and Health.

No. 1606, Vol. LXI. SATURDAY, MARCH 17, 1917. PRICE ONE PENNY.

### WHAT IS TRUTH?

Our contemporary *Truth* has entirely reversed its policy since the death of its founder, Mr. Henry Labouchere. In his days the paper was a veritable *malleus hereticorum*, and exposed quackery and imposture of every kind, especially of the advertising and lucre-minting kind, with merciless severity. Many a libel action did Mr. Labouchere sustain from his enraged victims, and he seldom was cast in damages greater than the contemptuous sum of a farthing, though he was usually saddled with many expenses which the unsuccessful plaintiff could not pay. No doubt the actions paid him well as advertisements, and were not invited solely for the public good, but still they served the public good, and he was, in his own queer way, a public benefactor. Since his death *Truth* has discovered that there is another side to the account. Some of the advertising practitioners are still the victims of the editor's scathing exposures, but others he takes under his wing, and forwards their interests to the best of his ability, which is no doubt considerable, and all the more by contrast with his exposure of others. Thus, to put it in the vernacular, he has "gone bald-headed" in favour of a much-advertised system of mind-training, which, according to its director's account, bestows on its fortunate recipients never-failing memory, organising power, inventiveness, strong will, self-confidence, initiative, originality, tact, logical power, and many other desirable qualities, some of which are quite incommunicable, and others of which cannot be appreciably increased except by years of effort. The claims of the director are supported by flaming testimonials of precisely the same character as those which have supported other systems and discoveries and inventions which *Truth* has derided, and which have been made the butt of its gibes and scoffs and flouts and jeers. If any man is really fool enough to suppose that any system will enable him to acquire a never-failing memory, or strength of will, or originality, he and his money will soon be parted, and the director of the system will assist him in the transfer.

Another practitioner that *Truth* is now "boosting," to use the vernacular again, is Mr. Barker, the bone-setter. *Truth* is very indignant that medical men will not sit at Mr. Barker's feet and learn from him, or at the very least welcome him as an honoured colleague; and in especial that he is not allowed to occupy an official position amongst the practitioners entrusted by the War Office with the treatment of wounded soldiers. "The work he offers to do is that of a surgeon—the work of his life. . . . He is the foremost exponent of an art professed before him by other men, equally eminent, from whom he has learned. . . . Pride and prejudice forbid the acceptance of the offer. At the present time Mr. Barker in his private practice is extensively engaged in curing officers of injuries received on active service whom the medical profession cannot cure," and so on. In short, the envy and jealousy and professional pique of ignorant, stupid, hide-bound, unsuccessful, orthodox medical practitioners, who fail egregiously in curing their patients, are responsible for preventing the one genius who could cure them, and is anxious to do so, from having a chance to exercise his benevolent and beneficent functions. What a world! and what a profession! It makes the eulogist of mind-training burn with righteous fury and blaze with righteous indignation. There may, however, be another side to the matter, another motive for the opposition of medical authorities, if the opposition does indeed come from medical authorities, as it very likely may, though there is no proof that the opposition is not due to the unaided common sense of the non-medical authorities.

We hear much from his friends of Mr. Barker's successes! We do not hear of his failures. It is not to be supposed, it can scarcely be asserted, that he is always successful; and the application of methods which are admittedly violent, since they sometimes require the administration of an anæsthetic—the application of violent methods to injured limbs, if it is not successful, must be disastrous. Admitting, as we may admit for the sake of argument, that these methods are sometimes successful, the question arises whether it is worth while to gain these successes at the cost of other instances in which they are disastrous. Admitting, for the sake of argument, that the opposition to Mr. Barker's employment by the War Office does in fact come from doctors, it is possible that a non-medical authority who is possessed of ordinary common sense may say to himself: "I dare say Mr. Barker is sometimes successful, and that when he rushes in where surgeons fear to tread he may now and then bring off a sensational cure, yet I do know that the human body is a mechanism of infinite complication, and I cannot believe that a man who has passed no public test in anatomy can be uniformly successful, or often successful, in applying violence to an anatomical structure. I should as soon believe that a man, ignorant of the mechanism of a watch, could be uniformly or often successful in repairing an injured watch by stirring up its mechanism with a skewer. Mr. Barker may believe thoroughly in his own methods—no doubt he does—and he may have occasional successes—no doubt he has—but if I am told that his cures constitute so large a proportion of his attempts that he ought to be officially employed to treat wounded soldiers, I can only reply *Credat judæus.*" (*See page* 480.)

